# Paving the way to understanding female-headed households: Variation in household composition across 103 low- and middle-income countries

**DOI:** 10.7189/jogh.12.04038

**Published:** 2022-05-14

**Authors:** Ghada E Saad, Hala Ghattas, Andrea Wendt, Franciele Hellwig, Jocelyn DeJong, Ties Boerma, Cesar Victora, Aluisio JD Barros

**Affiliations:** 1 Department of Epidemiology and Population Health, Faculty of Health Sciences, American University of Beirut, Beirut, Lebanon; 2 International Center for Equity in Health, Federal University of Pelotas, Pelotas, Brazil; 3 University of Manitoba, Winnipeg, Canada

## Abstract

**Background:**

Female-headed households (FHHs) are regarded as disadvantaged. There are multiple social trajectories that can lead to women heading households. It is important to distinguish between these trajectories, as well as societal norms and contextual factors, to understand how and when are FHHs represented as a dimension of gender inequity. Our analysis defines and describes a typology of 16 FHH types (FHH16) based on demographic characteristics.

**Methods:**

This cross-sectional study used national Demographic Health Surveys (DHS) and Multiple Indicator Cluster Surveys (MICS) in 103 low- and middle-income countries (LMICs) to identify a typology of FHHs based on the family composition and additional household members. We performed descriptive analyses at the household level to generate median proportions of the FHH16 types and selected household characteristics. We conducted cluster analyses to explore FHH16 patterns across naturally grouped clusters of countries and described selected social and economic indicators at the ecological level.

**Results:**

The most common FHH16 types were those where the women household heads lived with children only, were alone, or lived with men, women, and children, but without a husband. In Africa and South Asia, the most common FHH was one where women heads resided with children only. In East Asia and the Pacific, the highest proportion of FHHs were those with men, women, and children. In MENA and Eastern Europe & Central Asia, households with women heads living alone were the most prevalent. Latin America had more FHHs with husbands, comparatively, and the most common FHHs were those with heads living alone or with children. Our exploratory cluster analysis generated five clusters with unique FHH16 patterns. The clusters had distinct geographic, contextual and economic characteristics.

**Conclusions:**

Our typology showed that FHHs are heterogeneous within and between countries. The ecological analysis emphasized further variation created by different societal and cultural factors. Research around their vulnerabilities and strengths needs to consider these factors and their influence on socioeconomic status and health-related outcomes within households headed by women.

There is a mainstream view in literature that female-headed households (FHHs) are among the most vulnerable households and are disproportionately present among the poorest of the poor [1-5]. However, this view that FHH are more disadvantaged than male-headed households (MHHs) has been challenged [1,2,6]; critics argued that FHHs are heterogeneous and that not all are poor, that the woman head tends to prioritize resource distribution towards household expenditure for better nutrition and health, and that poverty is multidimensional and can be interpreted subjectively [7]. Thus, there is a need to better understand women’s and children’s health-related behaviours and outcomes within these households.

Numerous trajectories lead to households being headed by women [7]. Married women may get separated from their husbands, or they may become widows and take responsibility for the household without re-marrying. Single women may go on to live alone, to be independent or for occupational reasons; single mothers may have to raise children alone. Married women may regard themselves as heads even with their husbands present, who may earn less or be disabled. These are possible trajectories leading to a FHH. Based on these varied trajectories, how FHHs fare in terms of socioeconomic status and health-related outcomes may vary considerably [5]. Also, the level of disadvantage faced by a FHH is greatly influenced by societal norms, contextual factors, and intra-household dynamics [2,8,9].

Patriarchal societies and households dictate gender roles and place constraints on women’s decision-making abilities, social mobility, workforce participation, and care responsibilities [10,11]. Patrilineal thinking stipulates that the man is the household’s main breadwinner and is responsible for its members, while the woman’s place is within the household, fulfilling the roles of wife, mother, and caregiver [10]. Moreover, the two-parent family structure is typically favored as the ideal family structure for children’s well-being [5]. When most households in a society follow these norms, households headed by women may be viewed as outliers and may be stigmatized. This could in turn adversely affect women’s and children’s mental well-being [6,12]. Furthermore societal norms may add obstacles to women’s participation in the workforce and limit the diversity of their jobs [10]. For women heading households, limited job opportunities and time constraints as a result of a triple burden of working, maintaining a household and caring for dependents, forces some women to take up more flexible jobs at the expense of earnings [13].

Most commonly, data on household structure and composition are predominantly collected in censuses and large household surveys, which rely on self-reported headship status [1,14,15]. The respondent subjectively assigns the household head and there are no predefined criteria for who may be regarded as such. This arbitrarily collected definition lacks an understanding of the respondent’s perspective of headship [3,5]. Its purpose is usually to administratively assure that all household members are accounted for by using the head as the reference person [14,15].

Alternatively, defining a FHH for research purposes may be based on the household’s demographic composition, considering whether the female head is married, and the presence of a husband, other adults and children in the household [5,15]. This categorization is based on an *a priori* definition and provides a level of distinction that allows for more nuanced categories of FHHs, yet generally, FHHs will be households where no men are present [5,15]. Another FHH definition is based on the economic contributions of each household member. In this approach, households are identified as being woman-headed in circumstances where the woman is the only or the main earner [1,5,15]. An FHH can also be defined based on decision-making power, where the household head is the person of authority in the household who exercises control over household resources and residents’ lives [1,5].

In this paper, we propose an FHH typology based on demographic characteristics informed by a theoretical background, using nationally representative household surveys. We then explore country patterns by applying this typology across world regions, and subsequently, across clusters with similar FHH type distributions, to consider what the underlying economic or cultural similarities within each cluster of countries may be.

## METHODS

### Data Sources

We used data from the cross-sectional Demographic Health Surveys (DHS) and the Multiple Indicator Cluster Surveys (MICS). These are nationally representative household surveys focused on health and nutrition among women and children (more information about these surveys is presented elsewhere [14,15]). From countries with DHS or MICS conducted since 2010, we selected the most recent one, including 103 low- and middle-income countries (LMICs). We used the UNICEF classification to categorize countries into seven world regions [16].

### Variables

We used the household and individual modules of the DHS and MICS, which are comparable in methods and indicators. To build our FHH typology, we selected variables from the household roster to categorize each individual’s relationship to the head of the household. We extracted variables on sex, age, and the member’s residency status (usual resident as opposed to visitors). This allowed use to define our households as female- or male-headed, in addition to household member composition.

To further describe the FHH typology, we generated the following variables from the DHS and MICS household module:

- Proportion of FHHs among the poorest 40% in the population.
- Woman household head’s average age.
- Proportion of FHHs residing in rural areas.
- Proportion of FHHs where the woman head had primary education or less.
- Proportion of women household heads who reported being currently married (only applicable to the 47 DHS surveys).

We used the household wealth index to identify the poorest 40% in the population, which is calculated separately for urban and rural areas based on household assets, building characteristics, water, and electricity availability among other variables. Subsequently the area specific scores are combined using a regression-based scaling approach [17].

For the ecological analysis that considers social and economic characteristics of the FHH types, we used the following indicators and data sources:

- *World Bank country income level:* four income groups – low (<$1035), lower-middle ($1036 and $4045), upper-middle ($4046 and $12 535) and high (>$12 536), based on the Gross National Income (GNI) per capita for the survey year [18].
- *Income inequality (Gini index)*: World Bank’s estimate [19]. The index ranges from 0 (perfect equality) to 100% (perfect inequality). The index closest to the survey year was used; 12 countries had missing indices.
- *UNDP’s Gender Inequality Index (GII):* includes three dimensions of gender-related disadvantage – reproductive health, empowerment, and labor market. The measure ranges from zero (gender equality) to one. GII values closer to one indicate higher inequalities between women and men [20]. The estimate closest to the survey year was used and 11 countries had missing indices.
- *UNDP’s Gender Development Index (GDI)*: measures the overall gender inequality in three dimensions of human development – health, education, and command of economic resources. Any absolute deviation from 1 (gender parity) means a deviation from perfect gender equality. Based on their deviation from parity, countries are defined as having high (<2.5%) medium-high (2.5-5%), medium (5-7.5%), medium to low (7.5-10%), and low (>10%) gender development equality [20]. The index closest to the survey year was used and five countries had missing indices.
- *Fragile States Index (FSI, The Fund for Peace Initiative):* consists of 12 qualitative and quantitative indicators around social, economic, and political pressures that provide an overall assessment of a country’s political and armed conflict risk. Indices at 0-29.9 refer to ‘sustainable’ countries, 30-59.9 are ‘stable’ countries, 60-89.9 are considered a ‘warning’ category, and an index of 90-120 means that the country is in the ‘alert’ category [21]. The index closest to the survey year was used and 4 countries had missing indices.

### Formation of the female-headed household typology based on a theoretical background

Based on the heterogeneity in social trajectories leading to the formation of FHHs, as well as the common societal norms and contextual factors that may play a role in the well-being of FHHs, we created a theoretical demographic typology using the data available in MICS and DHS. First, we sought to represent the family unit, characterized by the presence of the head, the spouse, and children, then exploring the presence of other adults in the household.

Demographic characteristics are collected in DHS and MICS in a household roster, including age and sex of each resident, starting with the head of the household as identified by the respondent. Our typology was constructed based on this information. All household residents younger than 18 years were classified as children, without differentiation between offspring of the head and others. Adult household members were those 18 years or older who were neither the head nor the spouse.

Households headed by females were categorized based on whether a husband, children, male adults or female adults were present. The resulting typology includes sixteen types of FHHs, illustrated in **Table 1**. Each type is represented by the letters “HMFC” denoting husband, other adult male, other adult female, and child, respectively. A capital letter indicates the presence of the member while a lower-case letter indicates absence. For example, hmfC represents a FHH where the woman head lives with children alone. We refer to this typology as FHH16 hereafter. Table S1 in **the** **Online Supplementary Document** articulates, in more detail, the sixteen types of the FHH16 typology.

**Table 1:** FHH16 – a female-headed household (FHH) typology with sixteen types based on demographic characteristics*

			Members of the family unit			
**Husband present**		**No husband present**	
**Children**† **present**	**No children**	**Children† present**	**No children**
**Other household members**	Other adult male present	Other adult females present	1 HMFC	5 HMFc	9 hMFC	13 hMFc
		No other adult females	2 HMfC	6 HMfc	10 hMfC	14 hMfc
	No other adult male present	Other adult females present	3 HmFC	7 HmFc	11 hmFC	15 hmFc
		No other adult females	4 HmfC	8 Hmfc	12 hmfC	16 hmfc

H – husband, M – other males, F – other females, C – children

*A capital letter indicates the presence of the member while a lower-case letter indicates absence.

†The children included here are all children in the HH younger than 18. Older children are regarded as other males or females in the household.

### Analysis

We carried out descriptive analyses at the household level to characterize the prevalence of FHHs out of all households by country and the median prevalence of FHHs by world region. We then calculated the proportion of the 16 types of FHHs in each country and presented the median proportions of the FHH16 types by region. The use of medians instead of means was prompted given the asymmetrical distribution of the FHH types across countries.

To identify similar country patterns of FHH16 across countries we carried out cluster analysis at country level. The countries in each cluster were as homogeneous as possible, while as different as possible from countries in other clusters. We employed the agglomerative hierarchical cluster analysis (HCA) with Ward’s method and squared Euclidian distance measure [22,23]. We explored multiple cluster groupings using the generated tree diagrams and presented FHH16 patterns in each cluster and described country social and economic characteristics by cluster. We performed pairwise correlations between the above-listed indicators. Those that had correlations above 0.4 were plotted against each other to explore the cluster and country patterns.

We also sought to understand characteristics of FHH types globally, by region and within clusters by generating the median proportions within each FHH16 type by country for the above-mentioned household and women heads variables.

All analyses were carried out using Stata (StataCorp. 2019. Stata Statistical Software: Release 16. College Station, TX: StataCorp LLC) and considered the survey sample design (clusters and sample weights).

### Ethics approval

This work is based on publicly available data from DHS and MICS. The ethical approval and consent were obtained by the agencies carrying out the surveys in each country.

## RESULTS

We studied 103 countries covering seven world regions. 22 countries were in West & Central Africa (WCA), 18 countries in Eastern & Southern Africa (ESA), nine countries in the Middle East and North Africa (MENA), 16 countries in Eastern Europe & Central Asia (EECA), seven countries in South Asia (SA), 11 countries in East Asia & the Pacific (EAP), and 20 countries in Latin America and the Caribbean (LAC). The survey years ranged from 2010 to 2019 and 445 708 FHHs were included in the analyses (out of 2 024 298 households). Study countries are listed in Table S2 in the **Online Supplementary Document****.**

**Figure 1:**
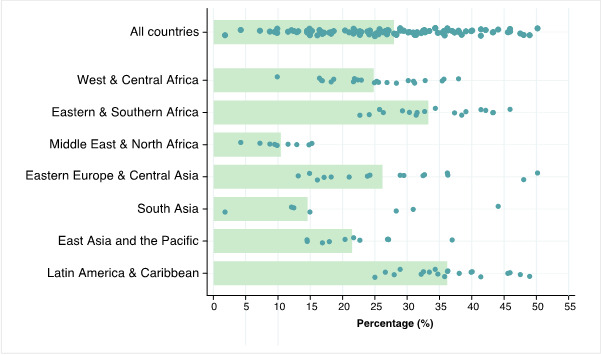
Median proportion of female-headed households (FHHs) out of all households, by UNICEF world regions and globally. Source: DHS and MICS, 2010-2019. *Each dot represents a country.

Globally, the median percentage of households headed by women was 28.0% and ranged from 1.7% in Afghanistan up to 50.1% in Belarus. Across world regions, the median FHH proportions varied from 36.2% in LAC to 10.5% in MENA (**Figure 1**, and Table S2 in **the** **Online Supplementary Document**).

**Figure 2:**
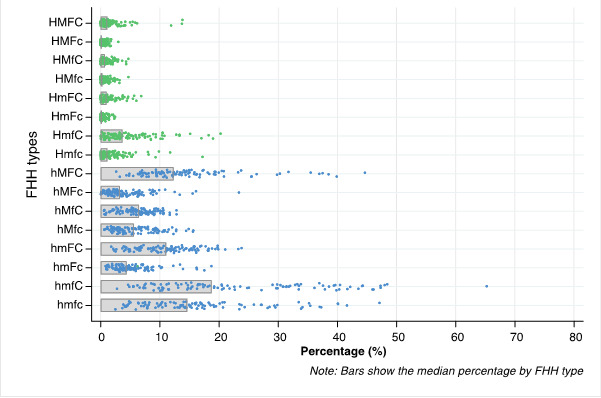
Distribution of female-headed households (FHH) by country among the FHH16 types. *H – husband, M – other males, F – other females, C – children Source: DHS and MICS, 2010-2019. *Each dot represents a country median, and the bars represent the median proportion of households within each FHH type across all countries. A capital letter indicates the presence of the member while a lower-case letter indicates absence.

**Figure 2** presents the median proportions of the 16 FHH types globally and by country. The most common FHH types were hmfC (18.7%), that is, those with a woman head with children only. Next, we had hmfc (14.6%) and hMFC (12.3%). These groups also presented the highest across-country variability. The median percentage of FHH with a husband present (H***) was only 9.5%, HmfC being the most common among those (3.6%). Generally, FHH types with children (***C) were more frequent than the corresponding type with no children (***c).

**Figure 3:**
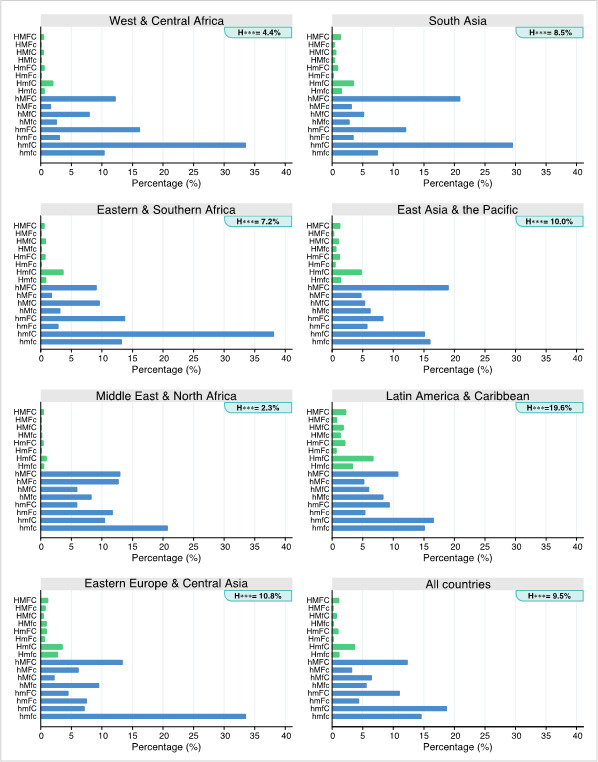
Median proportion of the 16 female-headed household (FHH) types by UNICEF world regions. Source: DHS and MICS, 2010-2019. H – husband, M – other males, F – other females, C – children. *Green bars represent FHH types, including a husband (H***) and blue bars, without husband (h***). H*** refers to the sum of the eight FHHs where a husband is present, with respective proportions. A capital letter indicates the presence of the member while a lower-case letter indicates absence.

**Figure 3** presents the median proportions of FHH16 types by world regions. Countries in WCA and in ESA showed similar patterns, where households with women heads with children only (hmfC) had the highest median prevalence, over 30%. The MENA region presented the lowest proportion of FHH with a husband (2.3%) while 20.7% of FHH were those with women living alone (hmfc). In EECA, the most common type was hmfc (33.6%.). In SA, hmfC was the most common group (29.5%), followed by hMFC (20.9%). LAC had the highest proportion of FHH with a husband (H***), especially with children present (HmfC = 6.7%). EAP was very similar to LAC.

The overall patterns of socioeconomic characteristics (wealth and head’s age) showed that FHH types least likely to fall in the poorest 40% were FHH with the head older than 50 years, without children and with a husband (Figure S1 in the **Online Supplementary Document**). The FHH types least likely to be in the poorest 40% households were HMfc (21.6%), HMFc (21.7%), hMFc (24.3%) and HMfc (25.4%). Conversely, the types most likely to be among the poorest were hmfC (40%-46.6%) and hmfc (46.1%). Households where husbands or other adults were present did not systematically translate into being less poor.

Examining the socioeconomic characteristics by region, the patterns differed to some extent, yet similarities were clear. Women heads were older in FHH with no children, and they were youngest where husbands and children were present. Also, the groups least likely to be among the poorest 40% were those without children and with a husband (Figure S3 in the **Online Supplementary Document**). On the other hand, the types most likely to be poor varied widely across regions.

In the cluster analysis to find groups of countries similar in terms of the FHH16 type distribution, we assessed three solutions – four, five or six clusters. We opted for the five cluster solution since the four and the six cluster solutions generated uneven distributions of countries within cluster, what made it difficult to interpret. Table 2 and Table S3 in the **Online Supplementary Document** describe the countries and their respective world regions within each cluster. Most clusters comprised countries predominantly from one or two regions. Clusters 1 (21 countries) and 2 (22 countries) mainly consisted of WCA and ESA countries. Cluster 3 included 28 countries, more than half from LAC and a quarter from EAP. Cluster 4 comprised mainly EECA countries, followed by MENA countries. Cluster 5 included 14 countries and was more diverse in terms of regions, the majority located in Asia.

**Table 2:** Summary of social, economic, and household indicators for each of the five clusters of 103 countries*

		Cluster 1	Cluster 2	Cluster 3	Cluster 4	Cluster 5
**Most frequent (>10%) median proportions of FHH16 (in descending order)**†		hmfC (43%)	hmfC (28%)	hmfc (15%)	hmfc (34%)	hMFC (31%)
		hmFC (13%)	hmFC (16%)	hmfC (15%)	hMfc (11%)	hmfC (18%)
		hmfc (13%)	hMFC (15%)	hMFC (14%)	hmFC (10%)	hmFC (13%)
			hmfc (13%)		hMFC (10%)	
**Predominant world regions – n = number of countries of a region in cluster (% of countries per region out of total in cluster)**		WCA – n = 10/22 (48%)	WCA – n = 8/22 (36%)	WCA	WCA	WCA – n = 4/22 (29%)
		ESA – n = 10/18 (48%)	ESA – n = 7/18 (32%)	ESA – n = 1/18 (4%)	ESA	ESA
		MENA	MENA-2/9 (9%)	MENA	MENA – n = 5/9 (28%)	MENA – n = 2/9 (14%)
		EECA – n = 1/16 (4%)	EECA	EECA – n = 1/16 (4%)	EECA – n = 12/16 (67%)	EECA – n = 3/16 (21%)
		SA	SA – n = 1/7 (5%)	SA – n = 2/7 (7%)	SA	SA – n = 3/7 (21%)
		EAP	EAP – n = 2/11 (9%)	EAP – n = 7/11 (25%)	EAP	EAP – n = 2/11 (14%)
		LAC	LAC – n = 2/20 (9%)	LAC – n = 17/20 (61%)	LAC – n = 1/20 (5%)	LAC
**Country Income level (GNI per capita)**	**Median Description**	**LIC**	**LMIC**	**UMIC**	**UMIC**	**LMIC**
	**Median**	$740	$1795	$5300	$4635	$1340
	**Country variation (IQR)**	$550-$1090	$1440-$3570	$3240-$7665	$3560-$8430	$850-$3960
**Gini Index**	**Median**	43%	43%	43%	33%	35%
	**Country variation (IQR)**	39%-45%	36%-49%	36%-48%	29%-36%	32%-37%
**Gender Inequality Index**	**Median**	0.55	0.56	0.42	0.27	0.54
	**Country variation (IQR)**	0.53-0.64	0.54-0.62	0.36-0.48	0.19-0.33	0.46-0.58
**Gender Development Index**	**Median**	0.87	0.91	0.97	0.97	0.82
	**Country variation (IQR)**	0.83-0.93	0.87-0.97	0.95-1.0	0.92-1.0	0.77-0.88
**Fragile States Index**	**Median**	Alert	Warning	Warning	Warning	Warning
	**Country variation (IQR)**	87-100	80-95	65-79	67-79	82-101
**% FHH over all households**	**Median**	29%	28%	35%	21%	17%
	**Country variation (IQR)**	22%-33%	18%-38%	27%-40%	15%-36%	13%-23%
**Age of women heads**	**Median**	49.22yrs	49.57yrs	52.46yrs	54.73yrs	52.63yrs
	**Country variation (IQR)**	43.71-54.75	43.03-54.79	46.21-56.83	46.56-61.26	45.40-57.29
**Proportion of FHH among poorest 40% of the pop.**	**Median**	0.35	0.35	0.37	0.34	0.28
	**Country variation (IQR)**	0.23-0.48	0.24-0.46	0.26-0.45	0.25-0.46	0.14-0.40
**Proportion of FHH residing in rural areas**	**Median**	0.63	0.5	0.39	0.25	0.39
	**Country variation (IQR)**	0.41-0.77	0.36-0.70	0.22-0.60	0.12-0.26	0.19-0.60
**Proportion of women heads with no or primary education**	**Median**	0.81	0.7	0.47	0.26	0.73
	**Country variation (IQR)**	0.67-0.91	0.51-0.84	0.27-0.67	0.05-0.54	0.37-0.83
**Proportion of women heads currently married among FHH without a husband‡**	**Median**	0.21	0.17	0.15	0.08	0.24
	**Country variation (IQR)**	0.13-0.33	0.10-0.24	0.07-0.27	0.04-0.15	0.14-0.42

IQR – interquartile range, WCA – West and Central Africa, ESA – Eastern and South Africa, MENA – Middle East and North, EECA – Eastern Europe and Central Asia, SA – South Asia, EAP – East Asia & the Pacific, LAC – Latin American and the Caribbean Africa, LIC – low-income country, LMIC – lower-middle income country, UMIC – upper-middle income country, HIC – high income country, GII – gender inequality index, GDI – gender development index, FSI – fragile states index, FHH16- female-headed household typology, H – husband; M – other males; F – other females; C – children

*Source: DHS and MICS, 2010-2019

†A capital letter indicates the presence of the member while a lower-case letter indicates absence

‡This indicator is only present for 47 countries with DHS survey.

**Figure 4:**
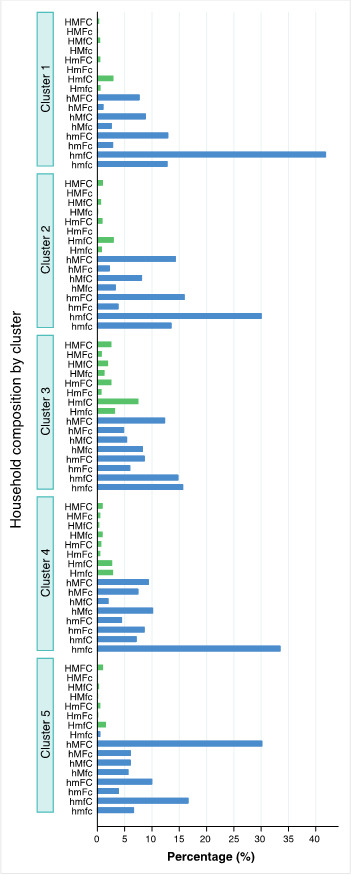
Median proportion of female-headed households (FHH16) typology within each of the 5 clusters for 103 countries. Source: DHS and MICS, 2010-2019 H – husband; M – other males; F – other females; C – children *A capital letter indicates the presence of the member while a lower-case letter indicates absence.

In **Figure 4**, we present the median proportion of FHH types for each cluster. Cluster 1 (mostly low-income African countries) had the highest proportion of hmfC type (40.3%). This is followed by FHH types hmFC (13%) and hmfc (13%). Cluster 2 (mostly African lower middle-income countries) was similar to Cluster 1, but with a lower proportion of household type hmfC (28.8%) and a higher proportion of hMFC (14.6%). Cluster 3 (mostly LAC and EAP upper-middle income countries) was unique in having the highest median proportions of FHHs with husbands, reaching up to 8.2% for HmfC types. It was also the most balanced in terms of all FHH types without the husband. The two most frequent FHH types in this cluster were hmfc (15.3%) and hmfC (14.7%). Cluster 4 (mostly EECA and MENA upper middle-income countries) was characterized by having the highest proportion of FHHs where the women were alone (hmfc = 33.5%) while the other FHH types without husband were relatively similar. Cluster 5 (Mix of lower middle income Asian countries) mainly had FHHs with an extended family without the husband (hMFC = 31.2%) followed by FHHs with children only (hmfC = 17.8%).

**Table 2** also presents a summary of the cluster characteristics in terms of country-level social and economic indicators as well as selected household characteristics. Figure S3 in the **Online Supplementary Document** illustrates the clusters’ median economic and social indicators. Clusters 1 and 2 consisted of mostly African countries, with low income, high income inequality, high gender inequality and high levels of country fragility. Cluster 2 was slightly richer than cluster 1. Clusters 3 and 4 have several aspects in common. These clusters were the richest ones, mostly including upper middle-income countries and presented better indices for gender development and less fragile countries compared to the other clusters. What differentiated them was income inequality, which was the highest in cluster 3 and lowest in cluster 4. Cluster 5 had mostly Asian countries with low income, low income inequality, high gender inequality and high state fragility. To give an idea of whether the patterns of FHH16 types depend on the percentage of FHHs within the country, we presented the median percentages of FHHs out of all households in the study population. Cluster 5 had the lowest median percentage of FHHs (17%), followed by cluster 4 with just over 20%. Clusters 1 and 2 were similar at around 28%. And cluster 3 had the highest percentage of FHHs at 35%.

**Figure 5:**
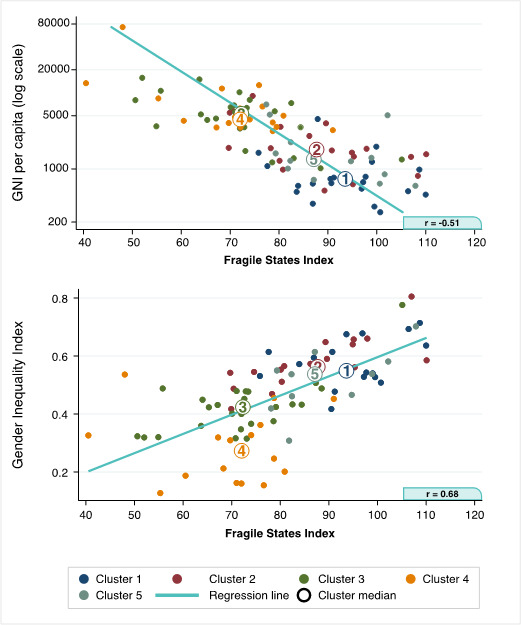
Country patterns and cluster medians by socio-economic indicators. Graph one illustrates the patterns by Fragile States Index and Gross National Income (GNI). Graph two shows the patterns by Fragile States Index and Gender Inequality Index.

In **Figure 5** we present country patterns and cluster medians by the socio-economic indicators that were well correlated with each other (r >0.4). In the top graph, we see that FSI and GNI were negatively correlated (r = -0.51) and that cluster 1 had the worst position with lowest median income level and highest state fragility. It was followed by clusters 5, 2, 4 and 3, with increasing income and decreasing fragility. In the bottom graph, GII was positively correlated with FSI (r = 0.68). Clusters 1,2 and 5 were very close together in a position of higher levels of gender inequality and fragility compared to cluster 3 (middle position) and cluster 4 (lowest inequality and fragility).

Regarding woman age (Figure S4 in the **Online Supplementary Document**), the type comprising the oldest women is hmfc in cluster 4. Again, generally, the types without a husband and without a child are the ones where the heads are older. In terms of being most likely to be in the 40% poorest household, the type hmfC consistently showed up in all clusters, followed closely by hmfc.

## DISCUSSION

In this paper, we introduced a detailed typology of FHHs using the most recent data from nationally representative household surveys in 103 low and middle-income countries. The proposed typology focuses on demographic characteristics of the households, generating 16 types of FHHs. This differentiation may provide insights into the socio-economic and health inequalities among FHHs where spouses are present; where only other adult males are present; where only adult females are present; or where both male and female adults are present. Our exploratory analysis showed there are distinct patterns of the 16 FHH types that cluster into natural groups with distinct contextual and economic characteristics. These distinctions may lead to different circumstances where women and children living in certain types of FHHs may be more vulnerable than those in other types of FHHs.

Our descriptive analysis of the FHH16 typology showed that the most common FHH types were households where the female head lived only with children (residents below age 18), or she was alone, or she lived with other adults (men and women) and children but without a husband. These findings are corroborated in literature, where thorough descriptions of FHH types show that a high proportion of the FHHs are those with lone mothers with children [5] and that the trend for extended households has declined in the past decade [5,9]. The high proportion of FHH being lone mothers may be in agreement with the idea that in the presence of a husband or senior male figure in the household, women are not likely to report being the heads irrespective of their role within the household.

While this was generally the case in most regions, there were some unique regional attributes. For example, in MENA countries, where the overall prevalence of FHH was the lowest (10.5%), the proportions of FHH types where a husband was present were negligible, while in LAC, the region that had the highest FHH prevalence (36.2%), a considerable number of FHH types had husbands present. Looking at the wealth profile in these two regions, it seems that presence of a husband did not translate into increased household wealth for LAC countries, unlike MENA countries. Having children in the household seems to have a bigger effect on poverty in LAC compared to MENA region.

The purpose of the cluster analysis was to see whether countries would group together based on similar patterns of FHH16 type prevalence that would be different from regional groupings, and to understand characteristics of these clusters that would help explain the patterns. Cluster 1 consisted of low-income African countries with high state fragility index. FHHs were almost 30% out of all households and women-headed households with only children present were most common (hmfC). These FHHs possibly had a high dependency ratio coupled with one source of income from the woman head [2]. It is possible that the husbands of these lone mothers have been involved in armed conflict in these highly fragile countries or have migrated abroad for better employment prospects [9]. In the latter case, they may have been sending remittances back to the household, which would have alleviated economic hardship [5]. Analysis by cluster showed that the hmfc (55%) and hmfC (51%) FHH types had the highest median proportions of being among the poorest 40% of the population. Yet, it is noted that the median proportion of heads currently married in hmfC was 45%, meaning that even if the woman head reported having a husband who was not a member of the household, his financial contribution may not have been substantial.

Cluster 2 is similar to cluster 1 in terms of Gini index, GDI, GII, and proportion of FHHs. Nevertheless, countries in cluster 2 tend to be richer and present a lower fragility index. In cluster 2 we found the highest proportion of women heads with children and another female (hmFC). The types hmFC and hMfC are common, too. Theoretically, this may mean that women heads were older in this cluster and the adults in the household were the women’s children. It may alternatively be that extended families were more common in these countries, leading to FHHs being better off because these members may contribute economically to the household expenditure [2,5]. Based on our analysis of household characteristics, hMFC women heads had one of the oldest median ages at 55 years. Compared to hmfC, hMFC had 20 percentage points less poverty, probably because the adults contribute to the household economically, as hypothesized.

Most of the countries in cluster 3 were from LAC, and upper-middle income countries predominate. The median Gini index was as high as clusters 1 and 2. Compared to clusters 1, 2, and 5, gender inequality was lower, gender development was almost at parity and country fragility in most of the countries was generally lower. This cluster had the highest prevalence of FHHs. It seems that in more peaceful, richer and more gender equitable countries, FHHs were more common and more equally distributed across types, including FHHs where the husband was present. It has been documented that the proportion of FHHs is relatively high in Latin American countries [4]. Cohabitation became common over time and may be linked to the presence of unstable relationships and possibly an increase in FHHs; moreover, women heading households in the presence of their husbands was relatively common [4].

Cluster 4 consists of Eastern European and Middle Eastern countries. It was similar to cluster 3 in terms of having the more peaceful and richer countries, but has lower Gini index and GII, and the proportion of FHH was considerably lower. FHH type hmfc, women alone, was by far the most common in the cluster. Considering insights gained from the MENA countries in this cluster, we may hypothesize that, even in the presence of high human development rankings, especially in oil-rich countries, there are cultural and religious norms that play an important role in shaping women’s lives and movements [10,24]. These norms may mean that not only females will not report as heads, but they will not accept women with children or older offspring living without a husband. Thus, FHHs are probably limited to women who were widows, often with grown up children with their own families, leading to the overwhelming proportion of FHHs with lone women [5,7]. Our finding that hmfc households in this cluster had the highest age median (65 years) and that they were the poorest is in agreement with this interpretation.

Cluster 5 mixes countries from a variety of regions, many from Asia. Country income levels varied considerably, the most common group being lower-middle income. This cluster had the lowest GDI, a high GII and lowest proportion of FHH. Along with cluster 1, it presented the worst state fragility profile. Our analysis showed that most households headed by women had men, women and children residing in them. Many of these Asian countries have patriarchal societal and religious norms [11,25] that may deter women from leading a household or reporting so, or even living without a man (husband, child or father). The FHHs that were present mainly have other adults and this adds to theory that women living alone were probably not socially accepted, cannot easily sustain a living, or need economic assistance from other adults [25]. In this cluster, mainly women living alone with or without children (hmfc and hmfC) had relatively high proportions of poverty.

Our proposed typology was standardized across a large number of LMICs using nationally representative large household surveys. This allowed us to compare patterns across countries and regions. Furthermore, our FHH typology was created using readily available demographic characteristics that may be adopted using different data sources. Nevertheless, our typology and subsequent analyses are not free of limitations. The typology uses self-reported FHH data and does not consider economic contribution to the household or decision-making power. Our data sources, ask respondents to indicate the household head without providing any guidance or criterion. Additionally, the indicated head must be a regular household resident. Consequently, the opportunity to create a more nuanced typology was limited [26]. The fact that data on household headship in these surveys is collected subjectively and without an *a priori* definition [26,27] means that some women, who may indeed be classified as heads under another definition of FHH (probably because they are the main breadwinners or the primary decision makers), may not report themselves as such due to societal norms that dictate that the man is the head of the household [28]. In literature, there are numerous definitions and categorizations of FHHs and no consensus on a given definition [1,27]. It is important to note that, based on how households headed by women are defined and identified, analyses of the household socio-economic characteristics and the health status in these households will potentially yield different results [5]. The resulting implications of using self-reported data on our analysis is reporting bias. This may mean that less FHHs are captured than there actually are (if a pre-specified definition of the FHH based on economic or decision-making criteria is adopted), or contrarily, it may mean that more households are identified as FHHs than there are (eg, if women are assigned as heads due to the lack of presence of the husband who may be working abroad). Regarding the analysis, the proportions of several FHH16 types were very low, especially among FHHs where husbands were present. Hence the household characteristics of rare types should be interpreted cautiously.

## CONCLUSIONS

In conclusion, our analysis has shown that FHH are not all the same and should not be treated as a homogeneous category; some are more vulnerable than others. Moreover, our ecological analysis emphasized further heterogeneity created by different societal and cultural factors. For researchers looking to study FHHs globally or within one country, it is important to understand how these factors may play a role in shaping FHH patterns and in influencing socioeconomic status and health-related outcomes within FHHs. Our FHH16 may contain types that are rare in some settings, but it opens the door to creating a more operational FHH typology by aggregating similar types based on our analysis and depending on the purpose of the research. It seems most useful for researchers aiming to explore women’s empowerment within FHHs, to compare FHHs with a husband present (with or without other adults and children), FHHs with no husband but with other adults (men and/or women) present and with or without children, FHHs where the women heads live with children only, and FHHs where the women heads are alone. For researchers studying child health and well-being within FHHs, the focus would be on FHHs with children only; they could be grouped into FHHs with husbands only, FHHs with other adults (men and/or women), and FHH with children only. In a linked study, we compared child health among male-headed households and two types of FHHs, FHHs where there are adult men present (husband or any other adult men), and FHHs where men are absent, to account for the low frequency of outcomes in several FHH types [29]. Further analyses and in-depth explorations will provide a clearer picture of which of the FHH types are more vulnerable, and which of them are in reality more efficient in how resources and spent and health is taken care of.

## Additional material


Online Supplementary Document
