# Supplementary material for: Paving the way to understanding female-headed households: Variation in household composition across 103 low- and middle-income countries
Source: J Glob Health. 2022 May 14;12:04038. doi: 10.7189/jogh.12.04038 (PMC9107795; doi:10.7189/jogh.12.04038)
Supplement: Online Supplementary Document [file jogh-12-04038-s001.pdf]

Paving the way to understanding female-headed households: variation in household composition across 103 low- and middle-income countries

Supplementary Material

Supplemental Table S1: Description of the sixteen types of female-headed households (FHH) that make up the FHH16 typology, using DHS and MICS surveys

FHH16 type	Description*
**HMFC**	FHH where the husband, other males, other females, and children are present
**HMFc**	FHH where the husband, other males and other females are present without children
**HMfC**	FHH where the husband, other males and children are present without other females
**HMfc**	FHH where the husband and other males are present without other females and children
**HmFC**	FHH where the husband, other females and children are present without other males
**HmFc**	FHH where the husband and other females are present without other males and children
**HmfC**	FHH where the husband and children are present without other males and females
**Hmfc**	FHH where the husband is present, only
**hMFC**	FHH where other males, other females and children are present without the husband
**hMFc**	FHH where other males and other females are present without the husband and children
**hMfC**	FHH where other males and children are present without the husband and other females
**hMfc**	FHH where males other than the husband are present only
**hmFC**	FHH where other females and children are present without the husband and other males
**hmFc**	FHH where other females are present, only
**hmfC**	FHH where children are present, only
**hmfc**	FHH where the female head lives alone

* Other males, other females and children may refer to one or several individuals of each category living in the household

Supplemental Table S2: Proportions of FHH types by the 103 countries and by UNICEF world regions. Source: DHS and MICS, 2010-2019

	Year	Source	Number of FHH households	FHH	HMFC	HMFc	HMfC	HMfc	HmFC	HmFc	HmfC	Hmfc	hMFC	hMFc	hMfC	hMfc	hmFC	hmFc	hmfC	hmfc
Benin	2017	DHS	3495	24.9%	0.3%	0.0%	0.4%	0.1%	0.8%	0.0%	3.3%	0.7%	9.2%	1.1%	8.1%	2.4%	15.8%	3.3%	40.1%	14.3%
Burkina Faso	2010	DHS	1507	9.9%	0.1%	0.0%	0.1%	0.0%	0.4%	0.0%	0.4%	0.1%	12.9%	1.2%	8.9%	1.6%	15.2%	3.0%	45.9%	10.4%
Cameroon	2018	DHS	3139	26.0%	0.7%	0.1%	0.5%	0.0%	0.6%	0.1%	1.7%	0.5%	14.1%	2.5%	7.5%	4.0%	16.6%	4.0%	30.7%	16.5%
Central African Republic	2010	MICS	2511	22.0%	0.3%	0.0%	0.1%	0.0%	0.5%	0.2%	2.9%	1.0%	10.6%	0.7%	7.6%	2.1%	17.0%	1.4%	41.7%	13.8%
Chad	2014	DHS	3852	22.1%	0.5%	0.0%	0.7%	0.1%	0.9%	0.1%	7.4%	0.5%	6.6%	0.5%	8.0%	1.3%	12.2%	1.2%	47.7%	12.2%
Congo Brazzaville	2014	MICS	3244	24.8%	0.0%	0.0%	0.3%	0.0%	0.4%	0.0%	2.6%	0.8%	10.9%	2.1%	5.2%	4.0%	20.0%	5.5%	30.0%	18.0%
Congo Democratic Republic	2017	MICS	5779	28.5%	0.2%	0.0%	0.4%	0.1%	0.4%	0.1%	2.9%	0.6%	12.1%	1.5%	7.5%	3.1%	15.5%	3.3%	41.9%	10.4%
Cote d'Ivoire	2016	MICS	2038	17.5%	0.2%	0.0%	0.0%	0.1%	0.2%	0.0%	0.5%	0.1%	14.1%	2.3%	6.5%	3.3%	21.2%	3.4%	32.8%	15.2%
Gabon	2012	DHS	3117	30.0%	0.9%	0.1%	0.4%	0.2%	0.6%	0.1%	1.2%	0.6%	15.5%	2.4%	8.2%	3.3%	15.7%	3.4%	26.8%	20.7%
Gambia	2018	MICS	1483	21.4%	1.7%	0.0%	0.7%	0.1%	1.4%	0.2%	1.7%	0.6%	29.3%	2.2%	8.1%	2.5%	23.4%	3.0%	18.7%	6.3%
Ghana	2017	MICS	4281	33.4%	0.7%	0.0%	0.5%	0.1%	1.2%	0.1%	3.1%	0.6%	10.9%	1.7%	9.1%	2.6%	20.1%	4.0%	35.8%	9.5%
Guinea	2018	DHS	1463	18.7%	0.7%	0.0%	0.5%	0.1%	0.4%	0.1%	1.9%	0.1%	21.0%	1.8%	8.8%	2.1%	23.1%	2.6%	31.4%	5.4%
Guinea Bissau	2014	MICS	1428	22.7%	0.4%	0.0%	0.0%	0.1%	0.5%	0.0%	0.0%	0.0%	37.4%	2.9%	11.2%	2.8%	20.2%	2.4%	17.6%	4.3%
Liberia	2013	DHS	3013	35.2%	3.4%	0.1%	4.0%	0.2%	5.8%	0.2%	19.4%	2.3%	12.3%	0.9%	5.5%	1.1%	12.4%	1.5%	24.9%	6.1%
Mali	2018	DHS	1741	17.4%	0.5%	0.0%	2.0%	0.2%	1.0%	0.0%	10.2%	1.0%	9.2%	0.7%	8.6%	1.4%	11.0%	1.7%	45.6%	7.1%
Mauritania	2015	MICS	4222	37.7%	2.3%	0.2%	1.2%	0.1%	1.7%	0.2%	8.3%	0.5%	13.3%	3.2%	7.4%	2.5%	19.4%	4.1%	31.5%	4.2%
Niger	2012	DHS	1627	15.9%	0.1%	0.0%	0.0%	0.0%	0.0%	0.0%	0.2%	0.0%	5.5%	0.6%	7.2%	1.3%	10.6%	1.3%	65.8%	7.5%
Nigeria	2018	DHS	7706	18.0%	0.1%	0.0%	0.0%	0.1%	0.1%	0.0%	0.6%	0.2%	7.0%	1.9%	7.0%	4.2%	13.4%	5.4%	34.2%	25.6%
Sao Tome & Principe	2014	MICS	1130	34.8%	0.5%	0.0%	0.5%	0.3%	0.1%	0.0%	2.0%	0.6%	5.8%	1.1%	9.9%	3.7%	13.0%	2.2%	44.9%	15.4%
Senegal	2017	DHS	2393	30.3%	2.1%	0.1%	0.8%	0.0%	1.2%	0.0%	1.6%	0.5%	39.8%	2.2%	6.6%	2.8%	20.2%	3.2%	14.3%	4.7%
Sierra Leone	2017	MICS	4803	31.3%	2.8%	0.2%	2.1%	0.4%	3.3%	0.1%	8.6%	1.1%	14.2%	1.2%	7.9%	1.5%	17.7%	2.0%	30.7%	6.1%
Togo	2017	MICS	2160	27.4%	0.3%	0.0%	0.2%	0.0%	0.5%	0.2%	0.8%	0.7%	7.2%	1.6%	9.6%	4.0%	14.9%	4.2%	37.0%	18.9%
**West & Central Africa MEDIAN**				**24.9%**	**0.5%**	**0.0%**	**0.4%**	**0.1%**	**0.5%**	**0.1%**	**2.0%**	**0.6%**	**12.2%**	**1.6%**	**8.0%**	**2.6%**	**16.2%**	**3.1%**	**33.5%**	**10.4%**
**West & Central Africa MEAN**				**25.0%**	**0.9%**	**0.0%**	**0.7%**	**0.1%**	**1.0%**	**0.1%**	**3.7%**	**0.6%**	**14.5%**	**1.7%**	**7.9%**	**2.6%**	**16.8%**	**3.0%**	**35.0%**	**11.5%**
Angola	2015	DHS	6145	34.5%	1.1%	0.0%	1.5%	0.1%	1.3%	0.1%	9.8%	1.5%	7.7%	0.9%	7.1%	2.0%	12.1%	1.7%	38.7%	14.5%
Burundi	2016	DHS	4613	28.7%	0.1%	0.0%	0.3%	0.1%	0.2%	0.0%	2.0%	0.1%	6.5%	2.0%	9.2%	3.0%	13.6%	4.3%	47.7%	10.9%
Comoros	2012	DHS	1761	39.3%	5.9%	1.0%	4.5%	1.9%	6.1%	0.7%	18.9%	2.9%	13.3%	2.6%	6.3%	1.6%	9.5%	4.1%	16.1%	4.6%
Eswatini	2014	MICS	2304	45.6%	0.1%	0.0%	0.1%	0.1%	0.2%	0.0%	0.7%	0.6%	16.7%	1.8%	12.4%	3.3%	17.8%	2.6%	29.5%	14.1%
Ethiopia	2016	DHS	5237	25.4%	0.4%	0.1%	1.0%	0.0%	1.1%	0.1%	4.4%	1.1%	7.7%	2.4%	9.6%	4.1%	9.8%	4.3%	38.8%	15.0%
Kenya	2014	DHS	12570	32.2%	0.3%	0.0%	0.5%	0.1%	0.3%	0.0%	2.1%	0.5%	7.7%	1.0%	10.8%	3.9%	12.8%	2.9%	39.2%	17.8%
Lesotho	2018	MICS	3625	41.1%	1.1%	0.0%	0.8%	0.1%	1.0%	0.1%	3.1%	0.8%	16.2%	2.3%	11.7%	7.1%	12.3%	3.8%	22.2%	17.4%
Madagascar	2018	MICS	3973	22.2%	0.7%	0.0%	0.7%	0.2%	0.5%	0.1%	4.8%	0.8%	8.7%	1.9%	10.3%	4.2%	11.3%	3.8%	37.5%	14.3%
Malawi	2015	DHS	7987	30.6%	0.5%	0.0%	0.9%	0.1%	0.8%	0.0%	4.3%	0.8%	6.9%	0.6%	12.2%	2.5%	12.0%	1.4%	48.8%	8.2%
Mozambique	2015	DHS	2862	38.0%	0.6%	0.0%	1.1%	0.1%	0.6%	0.1%	7.4%	1.6%	6.9%	0.8%	5.8%	1.9%	11.6%	1.0%	47.7%	12.8%
Namibia	2013	DHS	4184	43.9%	1.3%	0.1%	0.9%	0.2%	2.0%	0.0%	3.5%	1.8%	17.4%	1.9%	9.4%	3.3%	18.6%	3.3%	23.1%	13.1%
Rwanda	2014	DHS	3969	31.0%	0.1%	0.0%	0.2%	0.0%	0.2%	0.0%	1.4%	0.2%	8.9%	2.3%	11.1%	5.1%	16.5%	4.4%	40.2%	9.5%
South Africa	2016	DHS	4957	42.6%	0.6%	0.0%	0.6%	0.3%	0.7%	0.3%	1.4%	1.0%	15.6%	3.4%	9.6%	6.4%	16.1%	4.7%	20.7%	18.6%
South Sudan	2010	MICS	3992	42.0%	2.7%	0.3%	2.5%	0.2%	5.0%	0.2%	15.4%	1.1%	10.3%	0.8%	6.4%	1.2%	14.4%	1.2%	33.8%	4.6%
Tanzania	2015	DHS	3063	24.5%	0.7%	0.1%	1.3%	0.2%	0.9%	0.1%	3.8%	1.1%	12.6%	1.3%	9.4%	3.0%	13.9%	2.8%	32.9%	16.1%

Uganda	2016	DHS	6080	31.0%	0.3%	0.0%	0.7%	0.0%	0.7%	0.0%	3.8%	0.5%	7.7%	1.1%	9.2%	2.4%	13.9%	2.5%	43.8%	13.4%
Zambia	2018	DHS	3549	26.8%	0.3%	0.0%	0.5%	0.1%	0.7%	0.0%	2.1%	0.5%	13.7%	1.5%	10.0%	3.0%	16.4%	2.6%	37.0%	11.6%
Zimbabwe	2019	MICS	4319	37.7%	0.3%	0.1%	0.5%	0.1%	0.6%	0.0%	2.4%	0.5%	9.3%	1.8%	11.2%	3.6%	13.9%	2.3%	40.5%	12.9%
**Eastern & Southern Africa MEDIAN**				**33.3%**	**0.5%**	**0.0%**	**0.8%**	**0.1%**	**0.7%**	**0.1%**	**3.6%**	**0.8%**	**9.1%**	**1.8%**	**9.6%**	**3.2%**	**13.8%**	**2.8%**	**38.1%**	**13.2%**
**Eastern & Southern Africa MEAN**				**34.3%**	**1.0%**	**0.1%**	**1.0%**	**0.2%**	**1.3%**	**0.1%**	**5.1%**	**1.0%**	**10.8%**	**1.7%**	**9.5%**	**3.4%**	**13.7%**	**3.0%**	**35.4%**	**12.7%**
Algeria	2012	MICS	2815	10.5%	0.9%	1.1%	0.2%	0.2%	0.5%	0.1%	1.5%	1.1%	26.8%	23.8%	5.9%	10.0%	4.6%	11.7%	7.2%	4.2%
Egypt	2014	DHS	3680	12.9%	0.1%	0.0%	0.1%	0.0%	0.1%	0.0%	0.2%	0.2%	9.4%	7.8%	6.2%	15.8%	5.6%	8.3%	10.9%	35.2%
Iraq	2018	MICS	1833	8.9%	0.4%	0.1%	0.1%	0.0%	0.0%	0.0%	1.0%	0.6%	37.6%	12.7%	6.1%	8.3%	6.6%	6.9%	12.4%	7.2%
Jordan	2017	DHS	2268	12.2%	0.2%	0.1%	0.1%	0.2%	0.4%	0.0%	0.3%	0.0%	11.8%	13.3%	5.5%	13.1%	6.2%	17.9%	10.3%	20.7%
Qatar	2012	MICS	257	4.8%	1.1%	0.4%	0.0%	1.0%	2.4%	0.2%	7.3%	4.5%	6.4%	16.7%	1.1%	6.1%	5.9%	16.6%	6.7%	23.6%
State of Palestine	2014	MICS	962	9.2%	0.1%	0.0%	0.0%	0.0%	0.2%	0.0%	0.9%	0.5%	13.0%	10.1%	5.6%	7.1%	4.1%	18.1%	10.5%	29.8%
Sudan	2014	MICS	2288	14.2%	1.0%	0.1%	0.9%	0.3%	1.1%	0.2%	4.8%	1.2%	14.5%	4.7%	9.7%	3.8%	11.5%	5.7%	32.7%	7.9%
Tunisia	2018	MICS	1768	15.5%	0.1%	0.0%	0.1%	0.2%	0.1%	0.2%	0.2%	0.4%	8.6%	15.8%	2.0%	15.1%	4.9%	15.8%	10.1%	26.5%
Yemen	2013	DHS	1259	7.8%	1.7%	0.3%	0.2%	0.1%	0.6%	0.2%	0.8%	0.2%	20.1%	6.3%	7.8%	4.9%	12.3%	8.0%	20.0%	16.6%
**Middle East & North Africa MEDIAN**				**10.5%**	**0.4%**	**0.1%**	**0.1%**	**0.2%**	**0.4%**	**0.1%**	**0.9%**	**0.5%**	**13.0%**	**12.7%**	**5.9%**	**8.3%**	**5.9%**	**11.7%**	**10.5%**	**20.7%**
**Middle East & North Africa MEAN**				**10.7%**	**0.6%**	**0.2%**	**0.2%**	**0.2%**	**0.6%**	**0.1%**	**1.9%**	**1.0%**	**16.5%**	**12.4%**	**5.6%**	**9.4%**	**6.9%**	**12.1%**	**13.4%**	**19.1%**
Albania	2017	DHS	2451	17.3%	0.2%	0.1%	0.1%	0.1%	0.3%	0.1%	1.2%	1.1%	11.8%	6.1%	2.4%	12.2%	6.2%	9.5%	11.4%	37.4%
Armenia	2015	DHS	2574	33.2%	1.3%	0.9%	0.1%	1.2%	0.5%	0.4%	1.4%	2.8%	19.4%	10.6%	1.3%	7.8%	5.2%	8.5%	5.0%	33.7%
Belarus	2012	MICS	4045	50.4%	1.1%	0.8%	1.1%	2.8%	1.2%	1.7%	13.5%	17.0%	3.2%	1.9%	0.8%	9.3%	2.0%	3.7%	6.6%	33.4%
Bosnia & Herzegovina	2011	MICS	1109	18.9%	0.1%	0.4%	0.0%	0.9%	0.3%	0.8%	1.5%	2.9%	12.1%	7.8%	3.1%	11.7%	3.7%	8.1%	6.2%	40.4%
Georgia	2018	MICS	3672	32.0%	1.0%	0.6%	0.3%	0.9%	1.4%	0.7%	2.6%	2.2%	16.9%	9.8%	2.0%	11.4%	4.6%	10.0%	6.0%	29.6%
Kazakhstan	2015	MICS	5939	36.0%	1.7%	0.5%	0.7%	1.6%	1.3%	0.9%	5.3%	5.9%	14.6%	4.4%	2.1%	10.7%	5.4%	5.9%	10.1%	29.0%
Kosovo	2013	MICS	567	13.7%	0.0%	0.2%	0.0%	0.2%	0.0%	0.2%	0.2%	0.4%	44.0%	10.1%	2.9%	6.7%	4.5%	7.0%	8.0%	15.6%
Kyrgyzstan	2018	MICS	1979	29.3%	1.5%	0.1%	0.8%	0.5%	0.8%	0.3%	3.1%	2.6%	18.6%	3.6%	3.4%	9.5%	6.6%	6.2%	15.9%	26.5%
Moldova	2012	MICS	4187	36.0%	0.4%	0.3%	0.4%	0.8%	0.6%	0.9%	2.8%	4.2%	4.8%	3.0%	2.4%	9.1%	4.5%	5.3%	13.6%	47.0%
Montenegro	2018	MICS	774	23.6%	0.2%	1.2%	0.4%	2.0%	0.6%	0.6%	3.9%	2.4%	5.3%	7.2%	1.1%	12.5%	2.7%	13.7%	4.7%	41.5%
North Macedonia	2011	MICS	529	16.1%	0.9%	1.2%	0.7%	0.7%	0.9%	0.5%	1.5%	2.7%	14.7%	8.3%	1.3%	14.7%	1.9%	8.0%	4.2%	37.8%
Serbia	2014	MICS	1507	28.5%	1.3%	1.1%	0.7%	2.6%	1.1%	1.3%	5.1%	7.8%	7.6%	6.9%	0.6%	9.6%	2.5%	8.9%	3.1%	39.8%
Tajikistan	2017	DHS	1790	20.9%	4.4%	0.6%	1.4%	0.6%	1.0%	0.2%	4.6%	1.1%	37.8%	4.4%	3.9%	5.5%	7.3%	3.4%	15.8%	8.1%
Turkey	2013	DHS	1727	14.9%	1.2%	0.9%	0.4%	2.0%	1.0%	0.6%	4.8%	4.3%	9.4%	6.0%	3.7%	9.7%	4.4%	10.3%	7.7%	33.4%
Turkmenistan	2015	MICS	1513	24.0%	3.7%	1.1%	0.4%	0.8%	1.7%	0.7%	6.5%	1.5%	35.7%	6.2%	3.4%	8.0%	6.0%	4.2%	10.0%	10.2%
Ukraine	2012	MICS	5234	47.9%	1.6%	1.2%	1.0%	2.7%	1.4%	2.2%	8.1%	11.7%	5.7%	3.1%	0.8%	9.4%	2.8%	5.8%	6.4%	36.1%
**Eastern Europe & Central Asia MEDIAN**				**26.2%**	**1.1%**	**0.7%**	**0.4%**	**0.9%**	**0.9%**	**0.6%**	**3.5%**	**2.7%**	**13.4%**	**6.2%**	**2.2%**	**9.5%**	**4.5%**	**7.5%**	**7.1%**	**33.6%**
**Eastern Europe & Central Asia MEAN**				**27.7%**	**1.3%**	**0.7%**	**0.5%**	**1.3%**	**0.9%**	**0.8%**	**4.1%**	**4.4%**	**16.3%**	**6.2%**	**2.2%**	**9.9%**	**4.4%**	**7.4%**	**8.4%**	**31.2%**
Afghanistan	2015	DHS	396	1.7%	1.5%	0.0%	0.0%	0.0%	0.0%	0.0%	0.0%	0.0%	31.1%	5.1%	6.8%	1.7%	18.4%	3.5%	29.5%	2.3%
Bangladesh	2019	MICS	7308	12.7%	1.1%	0.2%	0.7%	0.3%	0.9%	0.2%	3.5%	1.5%	10.3%	3.0%	7.1%	5.6%	12.7%	4.1%	29.9%	18.8%
Bhutan	2010	MICS	4949	28.0%	11.8%	1.6%	4.9%	1.9%	6.9%	1.8%	16.5%	5.0%	15.9%	3.1%	3.3%	2.8%	6.4%	3.1%	7.5%	7.4%
India	2015	DHS	87381	14.6%	1.1%	0.3%	0.4%	0.4%	0.6%	0.2%	1.9%	0.8%	22.9%	8.0%	5.2%	9.4%	8.9%	4.6%	18.6%	16.4%
Maldives	2016	DHS	2818	44.3%	13.6%	2.3%	2.3%	1.3%	5.5%	0.8%	7.9%	3.8%	20.9%	3.2%	4.8%	2.6%	11.6%	2.2%	12.5%	4.7%
Nepal	2016	DHS	3589	31.3%	1.4%	0.5%	0.6%	0.4%	1.2%	0.4%	3.6%	1.8%	10.7%	1.2%	5.2%	2.6%	12.1%	3.0%	43.0%	12.1%
Pakistan	2017	DHS	1205	12.5%	0.9%	0.1%	0.0%	0.0%	0.2%	0.0%	0.3%	0.0%	25.0%	6.0%	8.2%	3.6%	15.3%	3.8%	32.2%	4.4%
**South Asia MEDIAN**				**14.6%**	**1.4%**	**0.3%**	**0.6%**	**0.4%**	**0.9%**	**0.2%**	**3.5%**	**1.5%**	**20.9%**	**3.2%**	**5.2%**	**2.8%**	**12.1%**	**3.5%**	**29.5%**	**7.4%**
**South Asia MEAN**				**20.7%**	**4.5%**	**0.7%**	**1.3%**	**0.6%**	**2.2%**	**0.5%**	**4.8%**	**1.9%**	**19.6%**	**4.2%**	**5.8%**	**4.1%**	**12.2%**	**3.5%**	**24.8%**	**9.5%**
Cambodia	2014	DHS	4277	26.9%	5.7%	0.9%	2.3%	0.7%	4.3%	0.8%	13.8%	2.4%	19.8%	4.7%	4.3%	4.1%	7.2%	5.2%	14.5%	9.2%
Indonesia	2017	DHS	7319	14.8%	0.2%	0.1%	0.1%	0.1%	0.2%	0.1%	0.5%	0.3%	18.7%	5.3%	5.4%	9.2%	8.6%	7.3%	14.5%	29.3%
Kiribati	2018	MICS	732	26.5%	13.0%	1.5%	2.7%	0.8%	3.4%	0.6%	10.8%	2.5%	30.5%	2.6%	5.2%	2.6%	6.4%	1.7%	11.8%	3.8%

Lao	2017	MICS	2781	13.9%	1.2%	0.3%	0.5%	0.4%	1.3%	0.5%	5.1%	0.8%	25.0%	6.8%	6.1%	6.5%	8.3%	5.7%	18.7%	12.7%
Mongolia	2018	MICS	2882	21.5%	1.0%	0.0%	0.7%	0.6%	0.9%	0.1%	4.1%	1.3%	11.8%	3.8%	5.4%	8.7%	11.3%	6.5%	19.8%	24.2%
Myanmar	2015	DHS	2869	22.5%	0.5%	0.1%	0.1%	0.2%	0.6%	0.2%	0.5%	0.5%	23.5%	9.2%	4.7%	5.9%	11.2%	13.1%	15.3%	14.5%
Papua New Guinea	2016	DHS	2709	17.5%	1.3%	0.0%	1.0%	0.1%	1.2%	0.0%	4.8%	1.4%	15.6%	2.2%	9.2%	3.5%	16.5%	3.1%	30.2%	9.9%
Philippines	2017	DHS	5363	20.6%	1.7%	0.3%	2.0%	0.6%	0.9%	0.5%	3.8%	1.5%	17.5%	6.5%	7.0%	10.8%	8.3%	7.4%	15.1%	16.1%
Thailand	2015	MICS	10836	37.3%	3.8%	1.5%	2.2%	2.4%	2.9%	2.1%	10.6%	8.4%	10.9%	5.6%	2.6%	6.7%	4.6%	8.3%	8.8%	18.7%
Timor Leste	2016	DHS	1995	17.5%	0.9%	0.0%	0.5%	0.1%	0.6%	0.1%	2.0%	0.4%	19.0%	3.6%	8.2%	6.2%	10.3%	5.5%	22.1%	20.5%
Vietnam	2013	MICS	2700	26.9%	4.7%	1.2%	1.8%	2.5%	3.2%	1.5%	11.4%	4.3%	19.7%	4.8%	2.7%	6.3%	3.9%	5.0%	8.8%	18.4%
**East Asia & the Pacific MEDIAN**				**21.5%**	**1.3%**	**0.3%**	**1.0%**	**0.6%**	**1.2%**	**0.5%**	**4.8%**	**1.4%**	**19.0%**	**4.8%**	**5.4%**	**6.3%**	**8.3%**	**5.7%**	**15.1%**	**16.1%**
**East Asia & the Pacific MEAN**				**22.4%**	**3.1%**	**0.5%**	**1.3%**	**0.8%**	**1.8%**	**0.6%**	**6.1%**	**2.2%**	**19.3%**	**5.0%**	**5.5%**	**6.4%**	**8.8%**	**6.3%**	**16.3%**	**16.1%**
Barbados	2012	MICS	1352	47.2%	1.8%	0.6%	1.3%	1.9%	2.0%	1.5%	3.9%	4.6%	9.9%	6.2%	6.1%	11.5%	9.5%	6.6%	9.3%	23.2%
Belize	2015	MICS	1511	33.0%	2.3%	0.7%	1.8%	0.9%	3.1%	0.6%	9.7%	4.2%	9.4%	3.1%	6.0%	5.2%	8.7%	4.3%	22.6%	17.5%
Colombia	2015	DHS	16244	36.4%	2.4%	0.7%	1.9%	1.6%	2.5%	0.8%	6.2%	3.2%	11.0%	6.0%	5.5%	9.7%	10.6%	7.5%	15.7%	14.6%
Costa Rica	2011	MICS	1835	36.5%	3.0%	1.6%	1.8%	1.3%	1.7%	0.9%	6.4%	3.1%	12.1%	6.3%	7.5%	10.5%	9.3%	7.0%	14.3%	13.2%
Cuba	2014	MICS	4744	48.5%	1.7%	1.6%	1.8%	4.6%	2.5%	2.4%	8.8%	10.0%	7.4%	6.1%	2.7%	11.0%	6.2%	7.1%	8.4%	17.7%
Dominican Republic	2014	MICS	10516	36.1%	1.8%	0.6%	2.1%	1.7%	2.1%	0.8%	8.5%	3.3%	8.3%	3.6%	8.6%	9.7%	9.3%	4.6%	20.9%	14.1%
El Salvador	2014	MICS	4066	34.6%	2.8%	1.0%	2.0%	1.1%	3.2%	0.7%	6.9%	1.7%	14.0%	5.4%	6.8%	8.2%	12.5%	6.4%	16.5%	10.8%
Guatemala	2014	DHS	5521	25.1%	1.4%	0.3%	0.5%	0.2%	1.0%	0.4%	1.9%	0.4%	15.2%	3.9%	7.9%	5.8%	15.8%	5.0%	32.3%	7.8%
Guyana	2014	MICS	1587	34.4%	2.3%	0.8%	1.8%	1.3%	1.2%	0.4%	4.3%	2.7%	15.3%	6.0%	8.4%	6.5%	11.0%	4.4%	16.7%	16.8%
Haiti	2016	DHS	5899	45.1%	3.2%	0.3%	2.8%	0.7%	3.0%	0.4%	9.0%	1.9%	14.1%	3.5%	8.8%	5.3%	14.3%	4.0%	21.6%	7.0%
Honduras	2011	DHS	5728	28.1%	1.6%	0.4%	1.7%	0.6%	1.8%	0.4%	2.9%	1.0%	17.6%	3.8%	9.7%	6.0%	14.1%	4.9%	25.2%	8.3%
Jamaica	2011	MICS	2774	46.2%	3.0%	0.4%	2.3%	1.8%	3.4%	0.5%	7.5%	3.6%	10.5%	3.0%	7.2%	6.7%	11.7%	4.8%	17.8%	15.9%
Mexico	2015	MICS	2390	26.5%	1.6%	1.0%	1.5%	0.9%	2.1%	0.6%	5.9%	2.6%	11.2%	7.1%	5.4%	9.8%	9.6%	8.3%	17.8%	14.6%
Panama	2013	MICS	2644	31.9%	2.9%	0.2%	1.6%	1.7%	2.6%	1.0%	7.6%	3.4%	10.6%	6.2%	6.6%	8.5%	8.3%	5.8%	17.4%	15.6%
Paraguay	2016	MICS	2721	38.7%	5.6%	1.1%	4.8%	1.5%	4.5%	1.2%	20.3%	5.8%	8.3%	3.2%	5.8%	5.7%	6.6%	4.3%	11.5%	9.9%
Peru	2018	DHS	8852	28.7%	2.2%	0.8%	1.4%	1.1%	1.5%	0.6%	5.2%	2.5%	10.0%	6.2%	5.3%	9.5%	9.5%	7.4%	20.3%	16.7%
St Lucia	2012	MICS	734	41.4%	1.3%	0.5%	2.2%	0.6%	2.1%	0.9%	5.4%	4.6%	10.5%	4.3%	5.6%	9.6%	7.4%	7.3%	14.1%	23.8%
Suriname	2018	MICS	3211	40.6%	5.1%	1.3%	3.0%	1.8%	3.4%	1.6%	8.7%	5.6%	13.7%	5.1%	5.0%	7.9%	8.7%	4.2%	10.9%	14.1%
Trinidad & Tobago	2011	MICS	1813	32.9%	1.2%	0.9%	0.9%	1.2%	1.3%	0.4%	3.5%	3.3%	12.7%	8.5%	4.6%	13.7%	8.7%	7.4%	11.1%	20.4%
Uruguay	2012	MICS	1408	40.5%	2.1%	0.6%	2.6%	1.5%	1.2%	0.3%	13.3%	9.8%	3.1%	2.7%	5.3%	7.5%	6.9%	4.4%	10.5%	28.4%
**Latin America & Caribbean MEDIAN**				**36.2%**	**2.2%**	**0.7%**	**1.8%**	**1.3%**	**2.1%**	**0.7%**	**6.7%**	**3.3%**	**10.8%**	**5.2%**	**6.0%**	**8.3%**	**9.4%**	**5.4%**	**16.6%**	**15.1%**
**Latin America & Caribbean MEAN**				**36.6%**	**2.5%**	**0.8%**	**2.0%**	**1.4%**	**2.3%**	**0.8%**	**7.3%**	**3.9%**	**11.3%**	**5.0%**	**6.4%**	**8.4%**	**9.9%**	**5.8%**	**16.7%**	**15.5%**
**GLOBAL MEDIAN**				**28.0%**	**1.1%**	**0.2%**	**0.7%**	**0.2%**	**1.0%**	**0.2%**	**3.6%**	**1.1%**	**12.3%**	**3.2%**	**6.5%**	**5.6%**	**11.0%**	**4.4%**	**18.7%**	**14.6%**
**GLOBAL MEAN**				**27.5%**	**1.7%**	**0.4%**	**1.0%**	**0.7%**	**1.4%**	**0.4%**	**4.9%**	**2.2%**	**14.5%**	**4.5%**	**6.4%**	**6.1%**	**10.9%**	**5.4%**	**22.8%**	**16.6%**
**TOTAL No. of Households**			**445,708**																	

Supplemental table S3: Distribution of countries & UNICEF world regions by clusters of similar FHH16 patterns.

**CLUSTER 1**					
Angola	*ESA*	Rwanda	*ESA*	Chad	*WCA*
Burundi	*ESA*	Uganda	*ESA*	Congo DR	*WCA*
Ethiopia	*ESA*	Zimbabwe	*ESA*	Mali	*WCA*
Kenya	*ESA*	Nepal	*SA*	Niger	*WCA*
Madagascar	*ESA*	Benin	*WCA*	Nigeria	*WCA*
Malawi	*ESA*	Burkina Faso	*WCA*	Sao Tome & Principe	*WCA*
Mozambique	*ESA*	CAR	*WCA*	Togo	*WCA*
**CLUSTER 2**					
Papua New Guinea	*EAP*	Zambia	*ESA*	Congo Brazzaville	*WCA*
Timor Leste	*EAP*	Guatemala	*LAC*	Cote d'Ivoire	*WCA*
Eswatini	*ESA*	Honduras	*LAC*	Gabon	*WCA*
Lesotho	*ESA*	Sudan	*MENA*	Ghana	*WCA*
Namibia	*ESA*	Yemen	*MENA*	Liberia	*WCA*
South Africa	*ESA*	Bangladesh	*SA*	Mauritania	*WCA*
South Sudan	*ESA*	Cameroon	*WCA*	Sierra Leone	*WCA*
Tanzania	*ESA*				
**CLUSTER 3**					
Cambodia	*EAP*	Barbados	*LAC*	Jamaica	*LAC*
Kiribati	*EAP*	Belize	*LAC*	Mexico	*LAC*
Indonesia	*EAP*	Colombia	*LAC*	Panama	*LAC*
Mongolia	*EAP*	Costa Rica	*LAC*	Peru	*LAC*
Philippines	*EAP*	Cuba	*LAC*	St Lucia	*LAC*
Thailand	*EAP*	Dominican Rep	*LAC*	Suriname	*LAC*
Vietnam	*EAP*	El Salvador	*LAC*	Trinidad & Tobago	*LAC*
Comoros	*ESA*	Guyana	*LAC*	Bhutan	*SA*
Kyrgyzstan	*EECA*	Haiti	*LAC*	Maldives	*SA*
Paraguay	*LAC*				
**CLUSTER 4**					
Albania	*EECA*	Moldova	*EECA*	Uruguay	*LAC*
Armenia	*EECA*	Montenegro	*EECA*	Egypt	*MENA*
Belarus	*EECA*	N.Macedonia	*EECA*	Jordan	*MENA*
Bosnia & Herzegovina	*EECA*	Serbia	*EECA*	Qatar	*MENA*
Georgia	*EECA*	Turkey	*EECA*	State of Palestine	*MENA*
Kazakhstan	*EECA*	Ukraine	*EECA*	Tunisia	*MENA*
**CLUSTER 5**					
Lao	*EAP*	Algeria	*MENA*	Gambia	*WCA*
Myanmar	*EAP*	Iraq	*MENA*	Guinea	*WCA*
Kosovo	*EECA*	Afghanistan	*SA*	Guinea Bissau	*WCA*
Tajikistan	*EECA*	Pakistan	*SA*	Senegal	*WCA*
Turkmenistan	*EECA*	India	*SA*		

Notes: ESA=Eastern & Southern Africa | SA=South Asia | WCA=West & Central Africa | EAP= East Asia & the Pacific | LAC=Latin American and the Caribbean | MENA=Middle East and North Africa | EECA= Eastern Europe & Central Asia

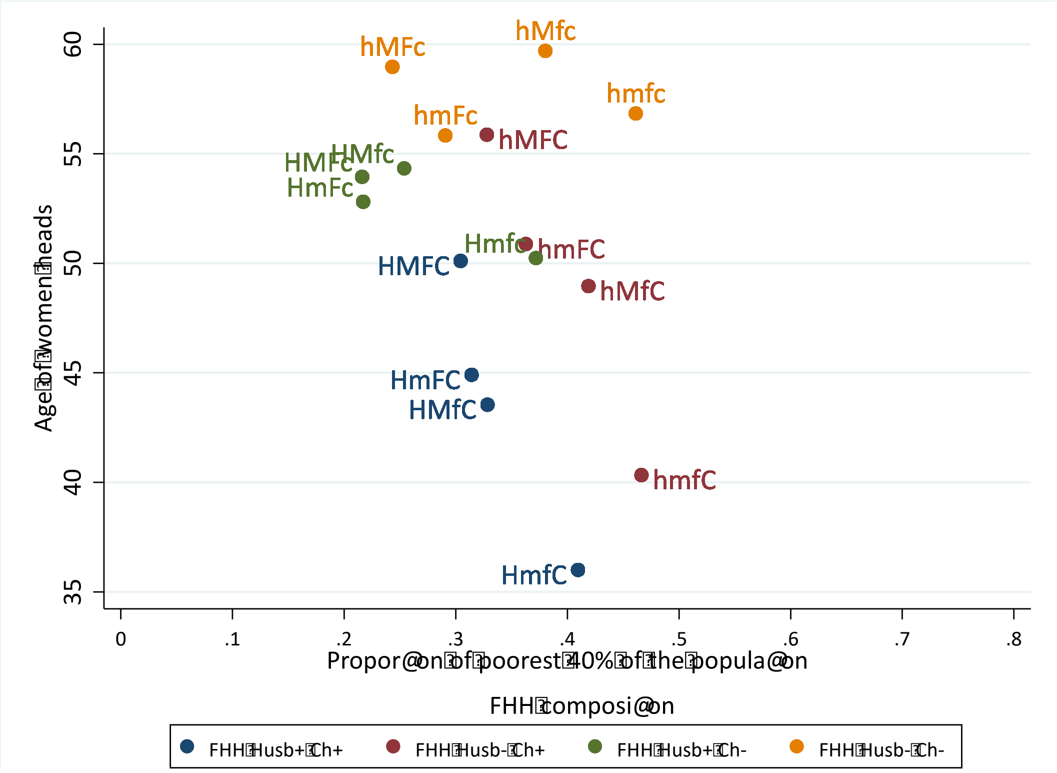

Supplementary figure S1: Median proportion of female-headed households among the poorest 40% of the population and age of women heads by FHH type across the 103 countries. Source: DHS and MICS, 2010-2019.

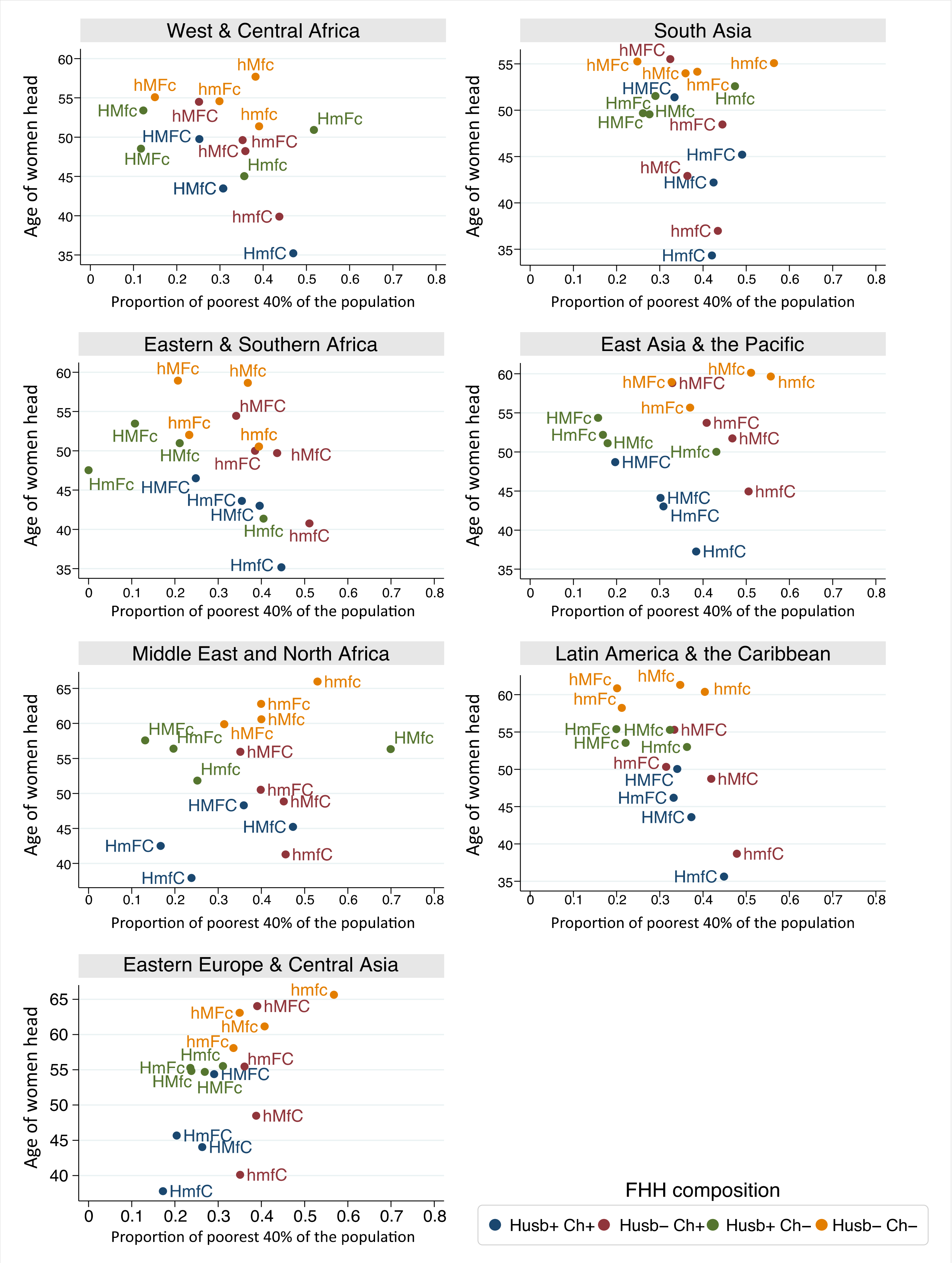

Supplemental figure S2: Median proportion of female-headed households among the poorest 40% of the population and age of women heads by FHH type across the 7 UNICEF world regions. Source: DHS and MICS, 2010-2019.

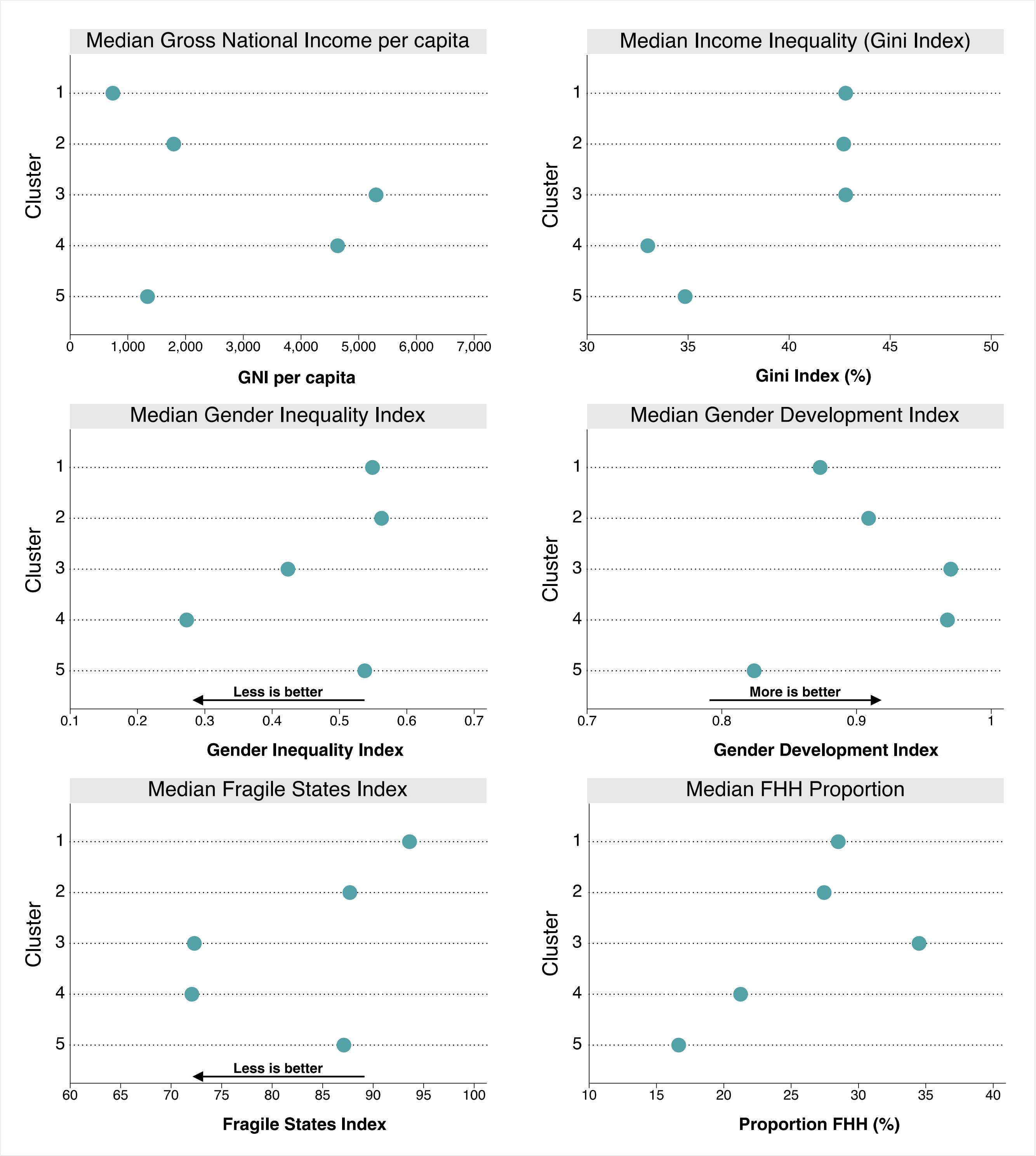

Supplemental Figure S3: Median proportions of social and economic characteristics within each cluster

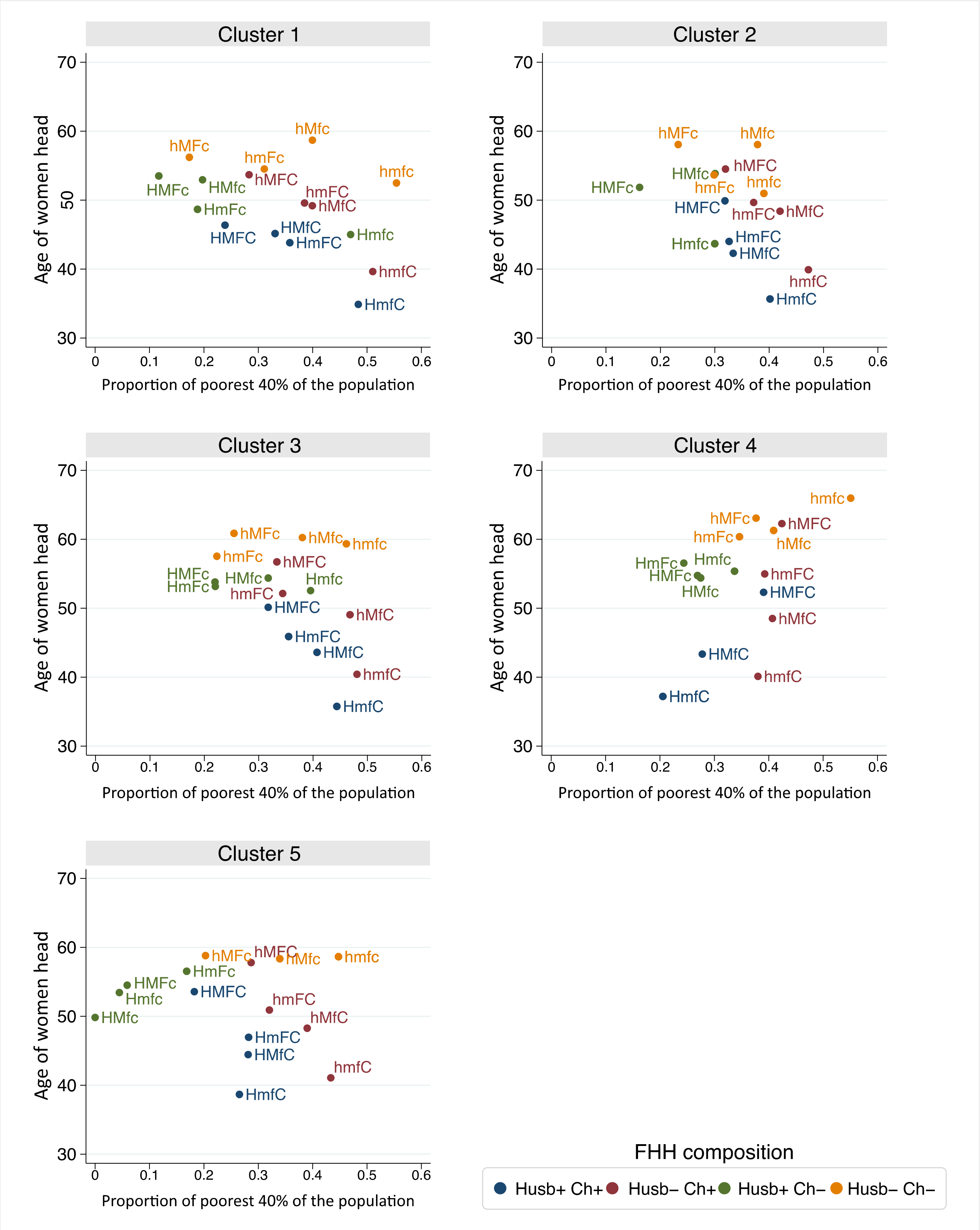

Supplemental figure S4: Median proportion of female-headed households among the poorest 40% of the population and age of women heads by FHH type across the 5 generated cluster. Source: DHS and MICS, 2010-2019.
